# Nutritional and Health-Related Effects of a Diet Containing Apple Seed Meal in Rats: The Case of Amygdalin

**DOI:** 10.3390/nu9101091

**Published:** 2017-10-02

**Authors:** Paulina M. Opyd, Adam Jurgoński, Jerzy Juśkiewicz, Joanna Milala, Zenon Zduńczyk, Bogusław Król

**Affiliations:** 1 Department of Biological Function of Food, Institute of Animal Reproduction and Food Research, Polish Academy of Sciences, Tuwima 10 Str., 10-748 Olsztyn, Poland; p.opyd@pan.olsztyn.pl (P.M.O.); j.juskiewicz@pan.olsztyn.pl (J.J.); z.zdunczyk@pan.olsztyn.pl (Z.Z.); 2 Institute of Food Technology and Analysis, Łódź University of Technology, Stefanowskiego 4/10 Str., 90-924 Łódź, Poland; joanna.milala@p.lodz.pl (J.M.); boguslaw.krol@p.lodz.pl (B.K.)

**Keywords:** amygdalin, apple seed meal, gastrointestinal tract, phloridzin, rat model, serum

## Abstract

Apple pomace includes seeds that are rich in protein, fiber, and oil, which can be extracted from them. However, they can also contain a significant amount of toxigenic amygdalin. We hypothesized that amygdalin is a compound that significantly reduces the nutritional and health quality of defatted apple seeds. An experiment was conducted on rats that were distributed into three groups and fed with high-fructose diets. In the control (C) and amygdalin (AMG) groups, cellulose and casein were the source of dietary fiber and protein, respectively; in the apple seed meal (ASM) group, dietary fiber and protein originated from the endosperm of apple seeds, which were previously defatted and ground. A diet fed to the ASM group also contained 0.24% of amygdalin from the meal, whereas the AMG diet was supplemented with the same amount of synthetic amygdalin. After 14 days of experimental feeding, the body weight of rats was decreased in the ASM group. When compared to the C group, apparent protein digestibility and nitrogen retention were increased in the AMG group, while both were decreased in the ASM group. In the small intestine, mucosal maltase activity was decreased in the AMG and ASM groups, whereas lactase activity was only decreased by dietary amygdalin. The caecal SCFA pool and butyrate concentration were significantly increased in the ASM group compared to the other groups. Moreover, the ASM diet increased plasma concentration of high density lipoprotein (HDL) cholesterol and plasma antioxidant capacity of water-soluble substances (ACW). It also decreased the liver content of thiobarbituric acid-reactive substances (TBARS). In contrast, dietary amygdalin did not affect these indices. Dietary supplementation with apple seed meal can exert beneficial effects on the intestinal tract, blood lipid profile and antioxidant status of rats. In most cases, these effects are not limited by the presence of amygdalin. However, the nutritional value of protein from apple seed meal is relatively low.

## 1. Introduction

The food industry generates large amounts of by-products, including those of fruit processing, which generally have less use and create environmental burden. However, a tendency toward increased utilization of fruit processing by-products, especially for the recovery of dietary ingredients or bio-active compounds, is growing worldwide [1]. Apples are one of the most popular fruit crops in the world. In 2014, total world production of apples was approximately 85 M tons, including 53 M tons produced in Asia, 17.5 M tons in Europe and 11 M tons in America [2]. Approximately 30% of global apple production is intended for the manufacture of juices and ciders, with pomace being the primary by-product [3]. Fresh apple pomace is usually composted and used as an ingredient in animal feed or dried and used for biogas production [4,5,6]. However, more and more efforts have been made to utilize this by-product in the food industry. For instance, apple pomace is a well-known source of pectin that can be easily recovered and used as a gelling, thickening, and stabilizing agent in many food products [7,8]. Apple pomace also contains a large amount of insoluble fiber: thus, it can be a part of fiber-rich supplements that regulate intestinal functions [9]. More recently, apple pomace has also been considered as a source of bio-active polyphenols, the most characteristic of which is phloridzin [10,11]. As with many plant phenolic compounds, apple polyphenols are thought to reduce risk for diet-related diseases such as cardiovascular disease, obesity, cancer, or diabetes due to their antioxidant properties, among others [12,13,14,15]. These actions are also related to phloridzin, which has been suggested as an effective agent in adjuvant treatment of obesity and hyperglycemia, primarily due to its inhibitory effect on glucose absorption from the small intestine [16].

An inherent part of apple pomace is seeds, the proportion of which varies from 2% to 3% of the dry material [17]. Apple seeds are rich in proteins, dietary fiber and lipids, which can mainly be found in their endosperm [18]. A relatively high content of lipids (approximately 29%) makes them suitable for oil extraction. Apple seed oil is rich in unsaturated fatty acids, especially linoleic acid, the proportion of which equals approximately 44% of its total fatty acids [19,20]. After extraction, considerable amounts of protein and fiber can still be found in seed residues for potential further use. However, apple seeds contain a significant amount of toxigenic amygdalin (1.0–3.9 mg/g seeds), a mandelonitrile gentiobioside classified as cyanogenic glycoside (CG). CGs are secondary plant metabolites that can be hydrolyzed in mammals to toxic hydrogen cyanide [21]. The toxic activity of cyanide is mainly based on its affinity to the terminal cytochrome oxidase in the mitochondrial respiratory pathway, which results in its blockade. As a consequence, cells are unable to use oxygen [22]. Thus, chronic consumption of amygdalin is considered dangerous and can disturb basic physiological functions in the organism. However, animal studies showing beneficial effects of amygdalin are also available in the literature. For example, intraperitoneally injected amygdalin to rats caused a reduction of renal fibrosis during chronic kidney disease progression [23].

The aim of this study was to determine whether meal obtained from the endosperm of defatted apple seeds containing amygdalin can be used as a dietary protein and fiber source. We investigated the effect of dietary apple seed meal or amygdalin by itself on nitrogen excretion patterns, intestinal functions, blood biochemical markers, and antioxidant status in rats that were fed a diet high in fructose and saturated fats. We hypothesized that amygdalin is an important compound that reduces the nutritional and health quality of defatted apple seeds. A high-fructose diet was used to induce metabolic disorders that are observed in diet-related diseases, such as dyslipidemia and oxidative stress, and thus to additionally check health-related effects of apple seed meal.

## 2. Materials and Methods

### 2.1. Preparation and Analysis of Apple Seed Meal

A fresh apple pomace produced as a by-product of commercial apple juice production (ALPEX Co., Łęczeszyce, Poland) was desiccated (70 °C). Seeds were subsequently separated and shredded. The obtained shreds were defatted in a Soxhlet extractor (Soxtec 8000, FOSS, Hillerod, Denmark) using hexane, initially dried at 40 °C (convective drying), and then dried in a vacuum at 70 °C. The defatted shot was ground in a ball mill, and the endosperm was separated from the husk using sifters to form apple seed meal.

Dry matter, total protein, and total dietary fiber were quantified in apple seed meal according to official methods of Association of Official Analytical Chemists (AOAC) intended for fruit products [24]. Amounts of amygdalin, benzaldehyde, and phloridzin in apple seed meal were determined by an HPLC method after three-step sequential extraction with 70% methanol. A chromatograph (Dionex system with UV detector, Germering, Germany) was coupled with Synergi^TM^ Fusion-RP 80Å column (150 × 2.00 mm, 4 μm, Phenomenex, Torrance, CA, USA). Gradient elution (flow rate 0.25 mL/min) was conducted using 0.05% phosphoric acid in water (phase A) and acetonitrile with 0.05% addition of phosphoric acid (phase B). Absorbance was measured at 210 nm for detection of amygdalin and benzaldehyde and at 280 nm for phloridzin detection. Details concerning the composition of apple seed meal are given in Table 1.

### 2.2. Animals and Diets

At the beginning of the experiment, 30 growing, male Wistar rats with similar weight (details in Table 2) were accommodated in individual metabolic cages in a temperature-controlled room (21 ± 1 °C) with relative humidity of 50% to 70% and a 12-h light/12-h dark cycle. Next, animals were randomly distributed into three groups of 10 rats each and fed with diets high in fructose and saturated fats for 14 days. All groups received semipurified diets with the same proportions of protein (10.1%), dietary fiber (5%), and fructose (68.75%). In the control (C) and amygdalin (AMG, positive control) groups, cellulose and casein were the source of dietary fiber and protein, respectively, whereas in the ASM group, dietary fiber and protein originated from apple seed meal. The diet fed to the ASM group additionally contained 0.24% of amygdalin from the meal, whereas the diet fed to the AMG group was supplemented with the same amount of synthetic amygdalin (Sigma-Aldrich, cat. no. A6005). Detailed composition of diets, which were freely available to rats for the entire experimental period, is given in Table 1. The use of rats was conducted in compliance with European guidelines for the care and use of laboratory animals and the animal protocol employed in this study was approved by the local Institutional Animal Care and Use Committee in Olsztyn, Poland (permission number: 23/2015).

### 2.3. Nitrogen Balance

At the midpoint of experimental feeding, nitrogen balance was determined. The 5-day preliminary period was aimed at adapting the gut microbiota to the diets, followed by a 5-day experimental period when feces and urine were collected once daily. Nitrogen levels in feces and urine were determined for each rat according to Kjedhal’s method. Apparent protein digestibility and nitrogen retention were used as criteria for nutritional quality of the diets. Apparent digestibility (%) of protein was calculated using the following formula:

$$APPARENT\;PROTEIN\;DIGESTIBILITY=\frac{protein\;intake-fecal\;protein}{protein\;intake}\times 100$$

Additionally, apparent nitrogen retention was calculated using the following formula:

$$APPARENT\;NITROGEN\;RETENTION=\frac{nitrogen\;intake-fecal\;nitrogen-urinary\;nitrogen}{nitrogen\;intake}\times 100$$

### 2.4. Collection of Biological Material and Analytical Procedures

After two weeks of experimental feeding, rats were anesthetized with sodium pentobarbital (60 mg/kg body weight). Each animal was weighed, and blood was subsequently collected from the tail vein. Serum was prepared by solidification and low-speed centrifugation (2500 *g* for 10 min at 4 °C). Then, selected internal organs (i.e., small intestine, cecum, kidneys, heart, and liver) were removed, weighed, and used for further investigation.

Disaccharidase activity (lactase, maltase, and sucrase) was assayed in jejunal mucosa samples by the method of Dahlqvist with modifications described previously [26]. Briefly, an aliquot of mucosal homogenate (0.1 mL) was incubated at 37 °C with 0.1 mL of substrate solution (sucrose, maltose, or lactose) in phosphate buffer (pH 7.0). After 30 min of incubation, cold distilled water was added, and the enzymatic reaction was interrupted by immersion of the test tube in boiling water for 2 min. In addition, a blank with the same composition was prepared and immersed in boiling water without prior incubation. Released glucose was quantified using a glucose oxidase reagent (Alpha Diagnostic Ltd., Warsaw, Poland). Disaccharidase activity was expressed as μmol of glucose liberated from disaccharide per minute per gram of protein. Mucosal protein concentration was determined using the Bradford method with bovine serum albumin as the standard.

Samples of fresh ileal and cecal digesta were removed, and the pH value, ammonia concentration, and dry matter content were then determined. Microbial enzyme activity and the concentration of short-chain fatty acids (SCFA) were determined after storage at −20 °C. The pH value of the intestinal digesta was measured using a microelectrode and pH/ION meter (model 301, Hanna Instruments, Amorim, Povoa de Varzim, Portugal). Ammonia concentration in the cecal digesta was determined in Conway dishes according to the method described by Hofirek and Haas [27]. Briefly, ammonia was extracted, trapped in a solution of boric acid, and then quantified by direct titration with sulfuric acid. Dry matter content of ileal and cecal digesta was determined at 105 °C. Microbial glycolytic activity in the cecal digesta (α- and β-glucosidase, α- and β-galactosidase, and β-glucuronidase) was measured by the rate of *p*- or *o*-nitrophenol release from nitrophenylglucosides according to the method described by Juśkiewicz et al. [28]. Enzymatic activity was expressed as μmol of product formed per hour per gram of digesta. SCFA concentration in the cecal digesta was analyzed by the means of gas chromatography (Shimadzu Co., Nakagyo, Kyoto, Japan) under conditions described previously [28].

Thiobarbituric acid-reactive substances (TBARS) were measured in heart, kidney and liver tissue after storage at −70 °C. A procedure developed by Botsoglou et al. [29] was used in the assay, and TBARS content was determined spectrophotometrically at 532 nm and expressed in μmol malondialdehyde per g of tissue.

Superoxide dismutase activity was determined in erythrocyte lysate using reagents from Randox Laboratories Ltd. (Crumlin, UK). The serum concentration of glucose, cholesterol (total and its HDL fraction) and triglycerides was determined using reagents from Alpha Diagnostics Ltd. (Warsaw, Poland). Serum ACW (antioxidant capacity of water-soluble substances, Analytik Jena AG kit, Jena, Germany) and ACL (antioxidant capacity of lipid-soluble substances, Analytik Jena AG kit, Jena, Germany) levels were determined with a photochemiluminescence detection method using a Photochem device (Analytik Jena AG, Jena, Germany), according to the method described by Popov and Lewin [30]. In the photochemiluminescence assay, generation of free radicals was partially eliminated by reaction with antioxidants present in serum samples, and the remaining radicals were quantified by luminescence generation. Ascorbate and Trolox calibration curves were used to evaluate ACW and ACL, respectively, and results were expressed as nmol of ascorbate or Trolox equivalent per mL of serum.

### 2.5. Statistical Analysis

All values are expressed as the mean ± standard error of the mean (SEM) (*n* = 10). Statistical analysis was carried out with STATISTICA version 6.0 (StatSoft Corp., Kraków, Poland). Data were tested by one-way analysis of variance and Duncan multiple range post hoc tests. A difference of *p* ≤ 0.05 was considered statistically significant.

## 3. Results

Although nitrogen intake during the 5-day balance period was similar among all groups, nitrogen content in feces and urine was higher in the ASM group and lower in the AMG group compared to group C (Table 2). These results inversely translated into differences in apparent protein digestibility and nitrogen retention, which were both lower in the ASM group and higher in the AMG group compared to group C. At the end of the experiment (day 14), dietary intake and body weight of rats were significantly decreased in the ASM group compared to the other groups. All animals survived the experimental feeding.

Physiological markers in the small intestine and cecum are shown in Table 3. Small intestinal mass and pH value as well as dry matter content of ileal digesta did not differ among groups. Maltase and lactase activity in the mucosa of the small intestine was decreased in the AMG and ASM groups, while sucrase activity was significantly decreased only in the ASM group, relative to group C. Furthermore, in the ASM group, the masses of cecal tissue and digesta relative to the body weight of rats were increased; in contrast, dry matter in the cecal digesta was decreased compared to the C and AMG groups. In cecal digesta, the ammonia concentration was decreased in both the AMG and ASM groups compared to the control group, whereas the pH value was lower in the ASM group than in the AMG group. Cecal β-glucosidase activity was decreased in the AMG group, whereas α-galactosidase activity was decreased in the ASM group compared to the C group. The dietary presence of both amygdalin and apple seed meal reduced β-glucuronidase activity, but the reduction was more significant in the AMG than in the ASM group. Dietary amygdalin and apple seed meal also affected SCFA formation in the cecal digesta (Table 3). The cecal SCFA pool and butyrate concentration were significantly increased in the ASM group compared to the C and AMG groups. Moreover, the concentration of branched chain SCFA (isobutyrate and isovalerate) was decreased by dietary apple seed meal (group ASM), while valerate concentration was increased by dietary amygdalin (group AMG) relative to the other groups.

Basic biochemical indices of blood serum were affected both by dietary apple seed meal and amygdalin (Table 4). Glucose concentration was decreased by dietary amygdalin compared to the C group. Triglyceride concentration was higher in the ASM group than in the AMG group. However, in both cases, it was comparable with the C group.

After 14 days of feeding, there were no significant differences in organ masses (liver, heart, kidneys) relative to body weight between groups (Table 5). TBARS concentration in liver tissue was decreased in the ASM group compared to the other groups. Serum ACW was increased in the ASM group compared to the other groups, while serum ACL was significantly higher in the ASM group than in the AMG group. Moreover, superoxide dismutase activity in erythrocyte lysate was significantly increased compared to the C group only by dietary amygdalin.

## 4. Discussion

The present study was conducted to determine whether a meal obtained from the endosperm of defatted apple seeds can be used as dietary protein and fiber source. The apple seed meal used in the current study was a good source of protein and fiber; these components equaled 55% and 27% of the dry matter, respectively. These quantities were considerably higher than those found in whole apple seeds by Kamel [31] (37% protein and 12% fiber) or in the endosperm of apple seeds (34% protein) by Yu et al. [18]. Bolarinwa et al. [32] have reported that the content of amygdalin in apple seeds can range from 1.0 to 3.9 mg/g. In the present study, however, amygdalin content determined in apple seed meal was several times higher (13.1 mg/g), which indicates that amygdalin remains primarily in the endosperm matrix during oil extraction. One of the primary products of amygdalin metabolism is benzaldehyde [33]. According to the literature, apple seeds contain less than 2 mg/g benzaldehyde [34], while in the current study, its content in the meal was 2.3 mg/g. In contrast, phloridzin is apparently less stable during apple seed processing because its content equaled only 0.6 mg/g of the meal, whereas in the study by Xu et al. [35], phloridzin content in whole seeds ranged 2.4–8.6 mg/g dry matter.

In the present study, rats from the AMG and ASM groups consumed approximately 171 and 148 mg of amygdalin daily per kg of initial body weight, respectively. These amounts were far from the lethal dose of 880 mg/kg body weight established by Adewusi and Oke [36]. Nevertheless, during two weeks of experimental feeding, control and AMG rats gained 17% and 20% of body weight, respectively, whereas ASM rats gained only 4%. These differences were mainly due to a significant decrease in food intake caused by dietary apple seed meal; this decrease was not, however, associated with dietary presence of amygdalin. We speculate that the main reason for lower dietary intake is an increase in intestinal production of SCFAs, which are known to stimulate satiety, possibly by activating free fatty acid receptors 2 and 3. These receptors modulate production of peptide tyrosine tyrosine (PYY) in enteroendocrine L-cells and leptin in adipocytes [37,38]. PYY and leptin act on the hypothalamus to reduce food intake, control energy expenditure and inhibit gastric emptying [39,40]. Another factor that could have stimulated satiety was the presence of phloridzin in the meal, which has anti-obesity effects related to suppression of sodium-dependent glucose transporter-1 (SGLT1) [16] and to the inhibitory effects of phloretin, the aglycone of phloridzin, on α-glucosidase activity [41]. Indeed, in the present study, the cecal SCFA pool was increased and mucosal sucrase activity was decreased by apple seed meal but not by dietary amygdalin itself. However, glucose concentration in blood serum was only slightly decreased by dietary apple seed meal, while dietary amygdalin decreased it significantly. Moreover, decreased apparent protein digestibility and nitrogen retention in the ASM group and the reverse pattern noted in the AMG group could also have affected body weight gain to a certain extent. In addition, the nitrogen balance results demonstrate that the nutritional value of protein from apple seed meal is much lower than that of casein; in contrast, amygdalin itself can increase protein digestibility and nitrogen retention in rats. Some authors suggest that benzaldehyde can inhibit pepsin activity and thus affect protein digestibility [42], whereas amygdalin can affect pancreatic protease activity, but this mechanism is not well understood [43,44].

The present study shows that both dietary amygdalin and apple seed meal can change the activity of endogenic and bacterial enzymes within the intestinal tract. In small intestinal mucosa, maltase activity was decreased by both dietary factors, while sucrase and lactase activity were decreased by meal and amygdalin, respectively. These findings suggest that the presence of amygdalin in experimental diets could be responsible for those changes. Indeed, hydrogen cyanide is known as a strong cellular respiration and enzyme inhibitor [22,45]. Interestingly, in the current study, lactase activity did not decrease in response to dietary apple seed meal, most likely due to the presence of phloridzin, which has been recognized as a substrate for this enzyme [46]. Moreover, a recent study by Kohl et al. [47] reported on seeds containing toxic glycosides and their inhibitory or stimulatory effect on the activity of endogenous and bacterial enzymes in spiny mice. These effects depended on the form of glycosides and adaptation of the organism. In the current study, enzyme inhibition was also noted in the cecum of rats; both bacterial β-glucosidase and β-glucuronidase activity were decreased by dietary amygdalin. In contrast, the meal did not exert the same inhibitory effect and was even able to stimulate α-galactosidase activity. An explanation of those differences may be the presence of fiber fractions in the meal; these fractions were potential substrates for bacterial enzymes. Importantly, a reduction in the activity of intestinal enzymes can benefit the host: for example, inhibition of mucosal sucrase and maltase activity can lower blood glucose levels [48,49]. A significant decrease was indeed noted in the AMG group, whereas inhibition of β-glucuronidase activity can reduce risk for colorectal cancer [50].

The ambiguous effect of amygdalin and apple seed meal on cecal activity of bacterial enzymes was reflected in cecal production of short-chain fatty acids. Total SCFA concentration did not differ between groups. However, the SCFA pool was increased in the ASM group, partly due to the lowered body weight of rats in this group. On the other hand, dietary apple seed meal increased cecal butyrate concentration, a preferred energetic substrate for colonocytes that appears to protect from colorectal cancer [51,52]. Apparently, cecal cells in the ASM group were better nourished, as cecal tissue mass was significantly increased compared to the other groups. Moreover, the concentration of branched SCFA (isobutyrate and isovalerate) was significantly decreased in the ASM group, which may suggest reduced putrefaction in the distal intestine, as branched SCFA are important metabolites of this process [53]. Another putrefactive metabolite is ammonia, which was also decreased after dietary apple seed meal. However, its concentration was also decreased in the AMG group, which suggests that the presence of amygdalin in experimental diets could be at least partially responsible for those changes. Nevertheless, the decrease in ammonia concentration is considered a positive change, because this compound can destroy cells, alter nucleic acid synthesis, and induce carcinogenesis in the lower bowel [54].

Dietary fiber (more specifically, its soluble fraction) is a well-known factor that regulates lipid metabolism, among other processes, by binding bile acids in the small intestine, thus lowering their re-absorption and increasing excretion with feces [55]. This is especially related to apple fiber, which primarily consists of pectin [56]. The present study showed that dietary apple seed meal had also favorable influences on blood lipid profiles by elevating HDL-cholesterol concentration. However, this effect did not depend on dietary amygdalin, which only slightly decreased triglyceride concentration (group AMG) compared to group C. That beneficial effect was principally related to the fiber fraction of the meal (27%). A small fraction of phloridzin (0.6 g/kg of the meal) could have also contributed to the effect, as this compound has been reported to improve lipid metabolism as well [16].

It is thought that oxidative stress is one of primary factors that leads to the development of chronic diseases such as diabetes and cardiovascular disease [57]. Superoxide dismutase is an antioxidant enzyme involved in the elimination of reactive oxygen species in the organism. In our study, dietary amygdalin significantly elevated superoxide dismutase activity. This elevation most likely was the organism’s response to oxidative stress induced by hydrogen cyanide. An in vitro experiment has shown that cyanides can induce oxidative stress and apoptosis in cells [58]. In contrast, in another study, superoxide dismutase was able to delay these processes [59]. Nevertheless, the antioxidative effect of apple seed meal was clearly shown in our study, since plasma ACW level was increased, and TBARS content in the liver, which is a marker of lipid peroxidation, was decreased. These results may be partly related to antioxidant effects of phloridzin, because this compound was shown to decrease the degree of lipid peroxidation in the liver of rats [60].

## 5. Conclusions

In conclusion, a defatted apple seed meal is a good source of protein and fiber. This study demonstrates that the nutritional value of protein from apple seed meal is relatively low, but that amygdalin itself can increase protein digestibility and nitrogen retention in rats. The daily dose of amygdalin standing at approx. 160 mg/kg body weight does not exert any observable adverse effects when it is fed to rats with a diet for two weeks. Dietary supplementation with apple seed meal can increase satiety or/and lower diet palatability and exert beneficial effects on the intestinal tract, blood lipid profile, and antioxidant status of rats. In most cases, these effects are not limited by the presence of amygdalin. Thus, despite the low quality of protein, defatted apple seed meal can be a valuable dietary factor for the prevention and treatment of diet-related metabolic disorders.

## Figures and Tables

**Table 1:** Composition of rat diets (%).

Diet Components	C	AMG	ASM
Casein ^1^	11.35	11.35	-
DL-methionine	0.15	0.15	0.15
Lard	7	7	7
Cholesterol	1	1	1
Cellulose	5	5	-
Apple seed meal ^2^	-	-	18.4
Amygdalin ^3^	-	0.24	-
Fructose	68.75	68.75	68.75
Corn starch	2.05	1.81	-
Mineral mix ^4^	3.5	3.5	3.5
Vitamin mix ^4^	1	1	1
Choline chloride	0.2	0.2	0.2
Calculated content			
Protein	10.1	10.1	10.1
Dietary fiber	5.0	5.0	5.0
Amygdalin	-	0.24	0.24

^1^ Casein preparation (g/100 g): crude protein, 88.70; crude fat, 0.3; ash, 2.0; water, 8.0. ^2^ Apple seed meal: dry matter, 94.02%; total protein, 54.63%; total dietary fiber, 27.21%; amygdalin, 13.1 g/kg; benzaldehyde, 2.3 g/kg; phloridzin, 0.6 g/kg. ^3^ Sigma-Aldrich, cat. No. A6005. ^4^ Recommended for the AIN-93G diet [25]. C: control group fed a high-fructose and saturated fat-containing diet; AMG: group fed a high-fructose and saturated fat-containing diet supplemented with amygdalin; ASM: group fed a high-fructose and saturated fat–containing diet with the addition of apple seed meal. “-“ refers to nothing.

**Table 2:** Dietary intake, nitrogen balance and body weight of rats.

Indices	C	AMG	ASM	ANOVA, *p* Value
Initial body weight, g	190 ± 6.0	191 ± 5.0	192 ± 3.5	=0.946
Nitrogen balance				
Nitrogen intake, mg/5 days ^1^	1152 ± 28.8	1186 ± 68.0	1160 ± 85.2	=0.491
Nitrogen in feces, mg/5 days ^1^	128 ± 2.6 ^b^	112 ± 9.7 ^c^	164 ± 18.1 ^a^	<0.001
Nitrogen in urine, mg/5 days ^1^	562 ± 51.0 ^b^	496 ± 64.5 ^c^	724 ± 52.1 ^a^	<0.001
Apparent protein digestibility ^2^, %	88.8 ± 0.43 ^b^	90.5 ± 0.98 ^a^	85.9 ± 0.94 ^c^	<0.001
Apparent nitrogen retention ^3^, %	40.0 ± 4.18 ^b^	48.8 ± 4.34 ^a^	23.4 ± 1.52 ^c^	<0.001
Dietary intake, g/14 days	187 ± 18.0 ^a^	191 ± 16.9 ^a^	166 ± 12.5 ^b^	=0.009
Final body weight, g	222 ± 22.9 ^a^	229 ± 15.9 ^a^	200 ± 7.4 ^b^	=0.001

All values are expressed as the mean ± SD (*n* = 10). Values not sharing the same superscript letters (^a, b, c^) within a row are different at *p* ≤ 0.05. ^1^ The 5-day preliminary period was aimed at adapting the gut microbiota to the diets, followed by a 5-day experimental period when feces and urine were collected once daily. ^2^ Apparent digestibility: (protein intake-fecal protein/protein intake) × 100; ^3^ Retention: (nitrogen intake-fecal nitrogen-urinary nitrogen/nitrogen intake) × 100. C: control group fed a high-fructose and saturated fat-containing diet; AMG: group fed a high-fructose and saturated fat-containing diet supplemented with amygdalin; ASM: group fed a high-fructose and saturated fat-containing diet with the addition of apple seed meal.

**Table 3:** Physiological markers of small intestine and cecum in rats.

Indices	C	AMG	ASM	ANOVA, *p* Value
Small Intestine				
Mass with digesta, g/100 g BW	3.31 ± 0.364	3.28 ± 0.325	3.522 ± 0.188	=0.165
pH of ileal digesta	7.20 ± 0.427	7.26 ± 0.378	7.12 ± 0.0.191	=0.665
Dry matter of ileal digesta, %	22.1 ± 2.28	21.1 ± 2.08	22.2 ± 1.17	=0.388
Mucosal disaccharidase activity ^1^				
Sucrase	20.1 ± 5.52 ^a^	17.8 ± 36.95 ^ab^	17.2 ± 3.54 ^b^	=0.035
Maltase	52.5 ± 5.55 ^a^	45.5 ± 8.97 ^b^	46.2 ± 1.68 ^b^	=0.032
Lactase	8.9 ± 2.38 ^a^	7.1 ± 3.05 ^b^	9.1 ± 3.42 ^a^	=0.010
Cecum				
Tissue mass, g/100 g BW	0.321 ± 0.041 ^b^	0.332 ± 0.079 ^b^	0.410 ± 0.116 ^a^	=0.049
Digesta mass, g/100 g BW	1.21 ± 0.344 ^b^	1.165 ± 0.284 ^b^	1.602 ± 0.473 ^a^	=0.027
pH of digesta	7.05 ± 0.147 ^ab^	7.11 ± 0.169 ^a^	6.94 ± 0.140 ^b^	=0.028
Dry matter of digesta, %	21.9 ± 3,17 ^a^	22.6 ± 2.08 ^a^	16.7 ± 2.68 ^b^	<0.001
Ammonia concentration, mg/g digesta	0.427 ± 0.020 ^a^	0.330 ± 0.064 ^b^	0.348 ± 0.042 ^b^	<0.001
Microbial enzyme activity ^2^				
α-glucosidase	6.71 ± 1.610	7.08 ± 1.105	7.24 ± 1.924	=0.751
β-glucosidase	2.13 ± 1.026 ^a^	1.13 ± 0.567 ^b^	2.61 ± 0.921 ^a^	=0.002
α-galactosidase	2.97 ± 1.052 ^b^	2.34 ± 0.544 ^b^	6.21 ± 1.160 ^a^	<0.001
β-galactosidase	37.9 ± 8.13	33.4 ± 8.68	31.0 ± 9.80	=0.236
β-glucuronidase	6.04 ± 1.274 ^a^	2.85 ± 0.638 ^c^	4.40 ± 1.813 ^b^	<0.001
SCFA concentration, μmol/g digesta				
Acetate	45.6 ± 10.27	46.6 ± 5.48	49.5 ± 9.94	=0.598
Propionate	10.0 ± 2.19	9.98 ± 1.03	11.2 ± 2.54	=0.357
Iso-butyrate	0.64 ± 0.26 ^a^	0.67 ± 0.23 ^a^	0.16 ± 0.04 ^b^	<0.001
Butyrate	5.34 ± 1.48 ^b^	5.49 ± 1.29 ^b^	8.05 ± 1.31 ^a^	<0.001
Iso-valerate	0.69 ± 0.21 ^a^	0.71 ± 0.18 ^a^	0.27 ± 0.06 ^b^	<0.001
Valerate	0.58 ± 0.23 ^b^	0.86 ± 0.16 ^a^	0.59 ± 0.14 ^b^	=0.003
Total	62.8 ± 12.82	64.3 ± 6.20	69.7 ± 13.19	=0.369
SCFA pool, μmol/100 g BW	72.8 ± 12.59 ^b^	73.9 ± 12.38 ^b^	108.0 ± 18.93 ^a^	<0.001

All values are expressed as the mean ± SD (*n* = 10). Values not sharing the same superscript letters (^a, b, c^) within a row are different at *p* ≤ 0.05. ^1^ μmol/min/g protein. ^2^ μmol/h/g digesta. SCFA: short-chain fatty acids; BW: body weight; C: group fed a high-fructose and saturated fat-containing diet; AMG: group fed a high-fructose and saturated fat-containing diet supplemented with amygdalin; ASM: group fed a high-fructose and saturated fat–containing diet with the addition of apple seed meal.

**Table 4:** Glucose concentration and lipid profile in the blood serum.

Indices	C	AMG	ASM	ANOVA, *p* Value
Glucose, mmol/L	13.1 ± 1.72 ^a^	11.1 ± 2.39 ^b^	11.5 ± 1.82 ^ab^	=0.040
Triglycerides, mmol/L	1.58 ± 0.157 ^ab^	1.18 ± 0.527 ^b^	1.76 ± 0.569 ^a^	=0.027
Total cholesterol, mmol/L	3.12 ± 0.675	2.84 ± 0.665	2.60 ± 0.390	=0.164
HDL cholesterol, mmol/L	1.29 ± 0.127 ^b^	1.23 ± 0.203 ^b^	1.44 ± 0.122 ^a^	=0.015
HDL cholesterol, % of total	41.0 ± 6.41 ^b^	43.0 ± 12.99 ^b^	55.0 ± 11.11 ^a^	=0.013

All values are expressed as the mean ± SD (*n* = 10). Values not sharing the same superscript letters (^a, b^) within a row are different at *p* ≤ 0.05. C: group fed a high-fructose and saturated fat-containing diet; AMG: group fed a high-fructose and saturated fat-containing diet supplemented with amygdalin; ASM: group fed a high-fructose and saturated fat–containing diet with the addition of apple seed meal.

**Table 5:** Markers of antioxidant status and oxidative stress in rats.

Indices	C	AMG	ASM	ANOVA, *p* Value
Blood				
Superoxide dismutase, U/mL	300 ± 48.1 ^b^	361 ± 41.9 ^a^	326 ± 21.1 ^ab^	=0.007
ACW, nmol/mL serum	51.7 ± 17.95 ^b^	62.2 ± 15.51 ^b^	81.2 ± 30.03 ^a^	=0.002
ACL, nmol/mL serum	53.2 ± 6.12 ^ab^	49.1 ± 4.51 ^b^	57.6 ± 5.91 ^a^	=0.007
Liver				
Mass, g/100 g BW	4.51 ± 0.558	4.53 ± 0.649	4.98 ± 0.374	=0.108
TBARS, nmol/g tissue	76.1 ± 0.72 ^a^	76.4 ± 0.63 ^a^	65.2 ± 0.85 ^b^	=0.003
Heart				
Mass, g/100 g BW	0.293 ± 0.026	0.278 ± 0.023	0.292 ± 0.013	=0.227
TBARS, nmol/g tissue	80.7 ± 1.36	85.6 ± 0.82	84.2 ± 1.01	=0.589
Kidneys				
Mass, g/100 g BW	0.655 ± 0.033	0.651 ± 0.038	0.677 ± 0.056	=0.381
TBARS, nmol/g tissue	127 ± 2.4	126 ± 1.9	124 ± 1.7	=0.937

All values are expressed as the mean ± SD (*n* = 10). Values not sharing the same superscript (^a, b^) within a row are different at *p* ≤ 0.05. ACW: antioxidant capacity of water-soluble substances; ACL: antioxidant capacity of lipid-soluble substances; TBARS: thiobarbituric acid-reacting substances; C: group fed a high-fructose and saturated fat-containing diet; AMG: group fed a high-fructose and saturated fat-containing diet supplemented with amygdalin; ASM: group fed a high-fructose and saturated fat–containing diet with the addition of apple seed meal.
